# Reflections on having children in the future—interviews with highly educated women and men without children

**DOI:** 10.3109/03009734.2012.654862

**Published:** 2012-08

**Authors:** Carola Eriksson, Margareta Larsson, Tanja Tydén

**Affiliations:** ^1^Department of Public Health and Caring Sciences, Uppsala University, Sweden; ^2^Department of Women's and Children's Health, Uppsala University, Sweden

**Keywords:** Attitudes, content analysis, fertility, parenthood, reproduction

## Abstract

**Background:**

There is a trend to delay birth of the first child until the age at which female reproductive capacity has started to decrease. The aim of the present study was to explore how highly educated women and men reflected on future parenthood.

**Methods:**

Twenty-two women and 18 men, who had started their professional career, were subjected to individual qualitative semi-structured interviews with qualitative content analysis guiding the analysis.

**Results:**

All informants, except for three women, planned to have children when some important prerequisites were fulfilled. Women and men reflected in much the same way, and prerequisites for parenthood were being of reasonable age and having a partner in the same phase of life. A reasonable age was considered in relation to reproductive capacity, and both women and men expressed awareness of the natural decline in fertility at higher ages. Good living conditions with stable finances were also important. Parenthood was perceived as a challenge and a sacrifice but also as enriching life. Reasons for having children included being part of the future and settling down to build their own family. Many concluded that there would never be a perfect time for having children.

**Conclusion:**

Highly educated women and men reflect on various factors when considering family planning. Being of reasonable age and having good living conditions, in particular a sound personal economy, were important. Given their goals, it is not surprising that many postpone parenthood until ages when female reproductive capacity is decreased.

## Introduction

In many European countries, women and men tend to postpone having children until ages at which their reproductive capacity has started to decline (1). In Sweden, the mean age at which women and men have their first child has increased by 4–5 years during the past 40 years and was 28.9 and 31.4 years, respectively, in 2010. As many as 12.6% of the nulliparous were 35 years or older. The mean age of first-time mothers varies in different parts of Sweden and is highest (32.4 years) in Stockholm, the capital of Sweden (2). The negative consequences of postponed parenthood are that some couples may not be biologically able to become parents, or to have the number of children they wish, combined with an increased risk for pregnancy complications. In addition, medical interventions and treatment of involuntary childlessness resulting from postponed child-bearing imply an economic cost for society and/or the couple and emotional distress for the individuals involved.

A range of factors are assumed to influence postponed child-bearing. In a review by Miles et al., effective contraception, increases in women's education and labour market participation, value changes, gender equity, partnership changes, housing conditions, economic uncertainty, and the absence of supportive family policies were pointed out as central reasons (3). The trend toward delayed home-leaving in Italy and Spain has also been shown to coexist with a very low fertility rate (4).

Sweden is well known for its long-standing supportive family policies such as generous paid parental leave and public child-care. These family policies seem to play an important role in keeping fertility relatively high (the total fertility rate in 2010 was 1.98) in spite of the trend toward postponing parenthood. Parental leave benefits are based on annual income from the preceding year. Having a stable income is therefore an important prerequisite for child-bearing. A group of young adults without a stable income is university students. Surveys of attitudes toward future parenthood and fertility awareness among undergraduate and postgraduate university students have shown that women and men had positive attitudes toward having children in the future, and the majority wished to have two or three children. As many as two out of three postgraduate female students wished to have their last child at the age of 35 years or older. One striking difference between the postgraduate students was that more women than men felt that having children while completing postgraduate studies was or would be difficult, and that parenthood would negatively affect their status on the labour market (5–7). Studies undertaken in Great Britain and Australia among women around 35 years of age have indicated that not having a partner and wanting financial security were the primary reasons for their childlessness (8,9). Most of the research on postponed parenthood has focused on women, but a recent study on male university students in Australia found that the men valued having a stable relationship and personal maturity before having children as well as completed studies, financial security, and a permanent and flexible job (10).

As delayed parenthood is common among highly educated people, and most research is based on standardized questionnaires or register data, the aim of the current qualitative approach study was to gain a deeper understanding of how highly educated women and men without children reflect on having children in the future.

## Material and methods

### Informants and study setting

We interviewed 22 women and 18 men who had at least 4 years of university education and had started their professional career at a work-place where the majority of the staff had a university education. The data collection was conducted in three university towns in Sweden: Uppsala, Umeå, and Stockholm. Permission for conducting the study was sought from the human resource manager at each work-place. Managers informed their staff about the study by e-mailing out our information letter, which described the aim of the study and stated that we wished to interview women and men, in their 30s, about their thoughts and reflections on having children. Those who were interested in participating were requested to phone or send an e-mail to the researchers for further information and arrangement of a suitable time for the interview.

### Data collection and instrument

The interviews took place in a quiet room offered by the work-place, or at a place outside the office. The interviews were audio-taped and lasted for about half an hour (range 20–60 minutes). Prior to the interview, all informants had been informed that participation was voluntary and that anonymity was guaranteed.

A semi-structured topic guide based on concepts derived from previous studies on university students was used (5–7). Topics covered in the interview were: personal attitudes toward having children in the future, timing of parenthood, and prerequisites for parenthood. Women and men were asked to reflect on the same questions. The interview started with questions on age, education, and civil status. Then all informants were asked: ‘Please, tell me your thoughts about having children in the future'. The answers given were then followed up by probing questions intended to promote clarification and expansion, for example ‘what do you mean when you say' or ‘what makes you think so'. The interviews were transcribed verbatim.

After the interview, the informants were invited to pose questions related to fertility issues to the interviewer.

### Data analysis

The interviews were analysed using content analysis (11). Interview transcripts were read carefully to get a sense of the whole. Then text was transferred into meaning units, which is a piece of text related to the aim of the study. These meaning units were condensed, i.e. shortening the text while still preserving the core content, and labelled with a code. All codes were sorted into nine subcategories and three categories according to their content and meaning. Transcripts and codes created from female and male informants were scrutinized separately and then compared. An example of the analysis process is given in Tables I and II. During this process all authors discussed the content of the subcategories and categories, and changes and redefinitions were made until consensus was reached. Translation of the quotes in the article was done by a professional Swedish–English translator.

**Table I.** Examples of meaning units, codes, subcategories, and categories.

Meaning units	Codes	Subcategory	Category
‘Children give a lot to you, happiness, joy, love … yeah, a lot' (w2)	Adds many positive qualities	An enriching of life	Pros and cons of family life
‘I think you learn a lot from having kids, that it helps you progress as a human being, you become more responsible and get a new perspective on what's important, and so on' (m2)	Increases responsibility and priority making		
‘It's a forever and life-changing decision, so it bears thinking about more than just once' (w19)	An irreversible commitment	A challenge	
‘I think that life with a child will be very demanding, I will likely be really tired and worn-out and doubt myself a lot, but at the same time, it will be really wonderful' (m9)	Both exhausting and wonderful		
‘But you're afraid of having to change the life-style you're used to, and of losing friends who don't have children and so are more active, while you yourself are locked in' (w16)	Personal forsaking	A sacrifice	
‘I understand people who choose not to have children, those who want to do as they please without having to compromise owing to the constraints that come with having a family' (m5)	Restricting	A sacrifice	

**Table II.** Subcategories and categories.

Subcategories	Categories
Being part of the future	Reasons for having children
Settling down to build their own family	
Adjusting to social expectations	
A stable relationship	Prerequisites for parenthood
Reasonable age	
Good living conditions	
An enriching of life	Pros and cons of family life
A challenge	
A sacrifice	

### Ethics

Verbal and written information regarding the aim and procedure were given to all participants. They were informed that they were free to withdraw from the study at any time and without declaring any reason. The study was approved by the regional ethical review board at Uppsala University, Sweden (No. 2010-085).

## Results

Participant characteristics for women (mean age 30 years, range 25–38) and men (mean age 30 years, range 24–36) are detailed in Tables III and IV. All but two were born in Sweden, and at the time of the interview two women and one man were expecting their first child.

**Table III.** Basic socio-demographic data on the participating women (*n* = 22).

Participant label	Age in years	Education	Civil status
w1	27	Economy	Single
w2	27	Economy	Cohabiting
w3	28	Pharmacology	Single
w4	28	Pharmacology	Single
w5	28	Economy	Cohabiting
w6	29	Economy	Married
w7	30	Biology	Cohabiting
w8	34	Political science	Cohabiting
w9	36	Political science	Cohabiting
w10	38	Pedagogy	Married
w11	28	Economy	Single
w12	25	Behavioural science	Cohabiting
w13	28	Medicine	Married
w14	26	Behavioural science	Single
w15	29	Technology	Cohabiting
w16	29	Medicine	Married
w17	34	Economy	Married
w18	29	Pedagogy	Married
w19	35	Biology	Cohabiting
w20	30	Biology	Cohabiting
w21	29	Technology	Single
w22	31	Medicine	Cohabiting

**Table IV.** Basic socio-demographic data on the participating men (*n* = 18).

Participant label	Age in years	Education	Civil status
m1	27	Technology	Cohabiting
m2	33	Medicine	Single
m3	36	Medicine	Married
m4	31	Economy	Single
m5	33	Medicine	Cohabiting
m6	24	Technology	Cohabiting
m7	26	Economy	Cohabiting
m8	27	Behavioural science	Married
m9	27	Political Science	Single
m10	30	Economy	Cohabiting
m11	30	Economy	Married
m12	30	Technology	Cohabiting
m13	30	Behavioural science	Married
m14	31	Technology	Single
m15	30	Economy	Cohabiting
m16	32	Biology	Single
m17	29	Biology	Cohabiting
m18	34	Medicine	Cohabiting

The vast majority of the informants wanted to have children, most commonly within the next 2–3 years. Three of the women had decided not to have children at all.

In the following section, the categories ‘Prerequisites for parenthood', ‘Reasons for having or not having children', and ‘Pros and cons of family life' describe how the informants expressed their reflections on having children in the future.

### Prerequisites for parenthood

Although many of the informants said that the ‘perfect' time for having children would probably never turn up, or that the ‘right' time could not be planned in advance, some prerequisites became obvious. The category ‘Prerequisites for parenthood' included three subcategories: ‘A stable relationship', ‘Reasonable age', and ‘Good living conditions'.

*‘A stable relationship'*, i.e. a relationship that had lasted for a couple of years, was to most of the informants an important prerequisite for having children. Nevertheless, some of the singles said that if they were to meet the ‘right' partner, they might be ready for children quite soon: ‘I think that when I meet the right one, then it might not take such a long time before I feel the urge to have children' (w4). The ‘right' partner was commonly described as ‘someone who shares one's interests and beliefs', ‘someone who can be trusted for the future', and ‘someone who is in the same phase of life'. The prerequisite of having a partner in the same life-phase was particularly emphasized by both the women and men. One man said: ‘We've been together quite a while now and we felt that it was time, seeing as we're both at that stage now' (m13). However, establishing a stable relationship was described by some as the main dilemma as regards having children, especially when living in a big city. One woman explained: ‘In a big city people are not as easily satisfied, they have very high expectations and it always seems like there might be someone better around the corner, and living together as partners is not such a high priority either, so finding the right partner is probably the biggest problem, as I see it' (w1). A man commented: ‘It's a problem you can really sense, I think, that everyone is continuously searching here and there for the “right partner”' (m14). In addition, the difficulty of establishing a stable relationship was also reflected on in relation to women's liberation. One woman explained: ‘You can manage just fine on your own, you have a fun life, and you earn good money, you don't need a partner for anything, really, so he has to be really good, otherwise you just can't bother with him' (w4).

*‘Reasonable age'* was considered important by the informants and often expressed as ‘not being too young or too old' (m5). Both ends were thought to constitute disadvantages, primarily for the child, but also for the parent. Being ‘too young' was seen as having too little experience of life, while being ‘too old' was thought to create generation gaps. One woman explained: ‘I don't regret not having had children at 22 because I've managed to do so many things for myself and I'm able to give my children so much more this way' (w1). A man said: ‘If you're approaching 40 then there will be a big difference between the age of the parents and the age of the child, and therefore you won't be as close to your child' (m12). It was also common, especially among the women, to relate the reasonable age of child-bearing to the age of one's own parents: ‘My mother was 29 when she had me and then she had my two sisters, so around 30 is a good age, I think' (w15), or to an expected, but unidentifiable ‘biological sign'. As an example one woman said: ‘So when is the right time, that's the eternal question, and everyone says “one day you'll just feel it's the right time”, but I can't say that I've felt that yet' (w9). Another woman explained: ‘I've always loved children and thought that I would have some of my own by this time, and that the older I got, the closer it would feel, like that the penny would drop, “Now it's time”, but in reality, it's still just as far away' (w2). In addition, the reasonable age was also considered in relation to reproductive capacity, and both women and men expressed awareness of the natural decline in fertility at higher ages, even though this was not set in advance. Another man said: ‘My wife will turn 34 soon, so we don't have so many years left, but we still feel like we can wait a couple of years' (m3), and one woman related: ‘I know that you are most fertile in your 20s, then it goes downhill, but I don't feel any pressure, but perhaps just before 35, maybe at 34, or at 33' (w3).

*‘Good living conditions'* were mentioned as crucial for having children, and a ‘healthy personal economy', i.e. ‘not having to save and scrape' (m6), was perceived as important, which required having ended student life and started a professional career. As an example one woman said: ‘Having reasonable finances, because that was something I experienced as a student, that there was never enough money, so at that time it was a relief not to have a child to provide for' (w10). For many of the informants, good living conditions also implied having good housing in a good environment. As an example one man said: ‘I would first, really prefer to find a roomy apartment or a child-friendly house' (m1), and another man explained: ‘We actually checked to make sure there were good child-care facilities and schools and so on before we moved' (m6). On the other hand, some expressed that even though these conditions were worth aiming at, they would not be decisive for their decision to have children. According to one man: ‘It's not very important to have a lot of money, but it's always helpful to have stable finances, so to speak, but if I wanted to have children without having all that, I would have them anyway, I don't think you should be overly worried about it, if you really want children, then it will work itself out' (m14).

### Reasons for having children

The category ‘Reasons for having or not having children' included three subcategories: ‘Being part of the future', ‘Settling down and build their own family', and ‘Adjusting to social expectations'.

*‘Being part of the future'* consisted of statements related to a wish to be part of and experience another human being's development, ‘The privilege of raising a child and participating in a child's growth' (w5). It also involved comments that revealed a desire to reproduce, to pass on one's heredity, familial ties, and beliefs, a desire more widely expressed by the men than by the women. As an example, one man explained: ‘The reasons I want to have children are to pass on my heritage, to be part of something together with my partner, and to pass on my knowledge and traditions' (m1). Another man stated that procreating was a human duty: ‘I want to have children and I think that if you can have children, you should have children, not having children is very selfish' (m4). On the other hand, one woman who had decided not to have children expressed the opposite view: ‘Wanting to have a little mirror image of yourself or the feeling that “now my legacy will live on because my genes will be preserved” is an egotistical undertaking that I just don't understand' (w8). In addition, having children was also thought to be the ultimate confirmation of the relationship with one's partner: ‘We felt that nothing more was needed for us to feel satisfied with our future life together' (w6), or simply as a desire to create life: ‘Personally, I find life to be such a joy, so I really want to pass it on to another human being' (w18).

*‘Settling down and build their own family'* involved reasons for having children that often were related to the informant's own upbringing. One woman said: ‘It has been wonderful for me to grow up with siblings, so the dream is to have a similar family of my own' (w1). Siblings were considered important to developing a sense of solidarity, and when creating one's own family unit two children were thought to be just right. One man explained: ‘You're not alone; you always have someone who is there for you and someone who is your best friend, and whom you can have fun with. So I think I'd like to have three children, though it will likely be two, because that feels more normal' (m2). It was also common to express a wish to be the same kind of parent one's own parents were: ‘I had a wonderful childhood, my parents created all the foundations for me to be successful in life, and I want to give my children the same opportunities' (m1). For both the women and men, a nuclear family was the foremost desire. One woman related: ‘I grew up with parents who are still married and I'm very satisfied with that, and I would like my children to have the same experience I had' (w5). However, alternative family constellations were also mentioned. One man said: ‘I have a number of friends who have children and are divorced and it seems to work alright, so I think that if you want to have kids then that's okay, even if it's not with your “one and only life-long partner”' (m14). Another woman said: ‘I have even calculated that I can make it on my own as a single mother, too, if it should come to that' (w4).

*‘Adjusting to social expectations'* consisted of reasons related to the life-style of people in one's immediate social environment. One woman said: ‘Not many of my friends or work-mates are interested in having children right now, though there is a difference in the type of discussions we have today as compared with a few years ago, at that time children weren't even a natural topic of conversation, so the thought of having children has become less distant now' (w3). And one man stated: ‘I come from a small town where a lot of my friends started having kids a long time ago, and I would surely have been the same way if I'd stayed there' (m14). ‘Adjusting to social expectations' also included statements revealing experiences of social pressure to have children. One married man described being under constant pressure at home: ‘I am constantly being nagged that it's “time” now, because my wife is very keen on having children' (m11). Some of the women described pressure related to social norms and expectations about what it means to be a woman: ‘You get those kinds of comments all the time, especially when you have a steady partner: “Why aren't you having children?”' (w7). In addition, having children was also considered a social milestone, i.e. ‘that you've succeeded with that goal in life, to have children' (w2), or as the way to finally become an adult: ‘You're looked upon as more grown-up when you've had children, I think' (m12). One of the women who had decided not to have children said: ‘It's the constant questioning and the feeling that there's something wrong with you because you don't want to have children, because a woman is expected to want children' (w10).

### Pros and cons of family life

Even if the vast majority of informants said that they wished to have children in the future, most of them also suggested that there were more than two sides to that coin. The category ‘Pros and cons of family life' included three subcategories: ‘An enriching of life', ‘A challenge', and ‘A sacrifice'.

*‘An enriching of life'* included comments revealing the expectations that having children would entail the addition of many positive qualities to one's life. One woman said: ‘Children give a lot to you, happiness, joy, love … yeah, a lot' (w2). And a man related: ‘Sharing the happiness of having a child with your partner, those around you, relatives and friends, I hope and believe that it will be a great joy' (m12). Having children was also perceived as something that helped one develop as a human being and increased one's sense of responsibility and priority setting. A man explained: ‘I think you learn a lot from having kids, that helps oneself to develop as a human being, you become more responsible and get a whole new perspective on life, a new outlook on what's important, and so on' (m2). Some said that having children may create positive behavioural changes and turn a person's attention away from oneself and toward another human being. As one woman explained: ‘I think that having children would have a very positive effect on me; that I would calm down a little and become more down-to-earth, and then, of course, that I would focus on someone other than just myself' (w5), and one man related: ‘You're not the prime consideration, but your needs come a distant second to someone else's, and I believe that aspect is helpful later on' (m5). In addition, having children was also reflected on as a turning-point that could change one's life, causing it to move in a new direction. As an example one man said: ‘Friends I talk to say that having children is the best thing that ever happened to them, it's even the case that those of them who had problems with various jobs and drugs and so on when they were younger have become completely different people since they had children, and you can see how happy they are' (m1).

*‘A challenge'* included statements indicating that most of the informants looked forward to having children. However, because parenthood is an irreversible commitment, the decision to have children was seriously considered. One woman explained: ‘It's a permanent and life-changing decision, so it bears thinking about more than just once' (w19). And one man said: ‘It's a big responsibility and you probably feel more alone today. There is no safety net if things go wrong, so you have to manage it on your own' (m7). The challenge of having children was also expressed in relation to perceptions about oneself as a parent, or what family life was supposed to be like. Another woman said: ‘I think I'm going to be a nervous wreck that won't get any sleep at all and that I'll get really frustrated because the child won't do what I want it to or what I think it should do' (w6). According to another man: ‘I think that life with a child will be very demanding, I will likely be really tired and worn-out and also doubt myself a lot, but at the same time, I think life with a child will be really wonderful' (m9). In addition, having children was also seen as a challenge for the relationship with one's partner. One man commented: ‘Having children could well put quite a strain on a relationship too; I've heard that nearly half of all parents of small children separate' (m17).

*‘A sacrifice'* consisted of comments revealing the perception that having children also entailed quite a lot of personal sacrifice, although this was more widely expressed by the women than by the men. One common concern was ‘losing freedom' (w1), often described in terms of being restricted from doing things that were perceived as difficult or in conflict with family life. As an example, one woman said: ‘We dream of going to Antarctica, of getting on a ship and looking at the animals, you can't do that when you have children' (w9). One man related: ‘I understand people who choose not to have children, those who want to be able to do as they please without having to compromise, owing to all the constraints that come with having a family' (m5). ‘A sacrifice' also implied concerns about giving up one's private or social way of living. Feelings of being afraid were expressed: ‘But you're also afraid of having to change the life-style you're used to, and perhaps of losing friends who don't have children and so are more active, while you yourself are locked in, which I understand is quite common' (w16). ‘A general fear of letting go of habitual life patterns and routines: I have to feel ready to go on to the next stage. Well, I bet I'll be a 33-year-old first-time mother' (w4). ‘A sacrifice' was also expressed in terms of ‘losing oneself'. One woman who had decided not to have children explained: ‘I have taken care of my nieces and nephews for extended periods of time and have experienced how you lose your sense of self, of being your own person, you don't have a thought for yourself, but are completely focused on what the child is doing and how to avoid the next disaster' (w8). In addition, ‘A sacrifice' also included concerns on the part of the women about damaging their body. One woman said: ‘Then it is a little awkward that so much depends on us women, even if it's a privilege too, but we're the ones who get pregnant, who will undergo these body changes and well, how will I look after child-birth?' (w15).

## Discussion

Our main finding was that these highly educated women and men in their 30s tried to balance their wish to have children in the future with important prerequisites such as good living conditions and a stable relationship, while avoiding being too old. They expressed thoughts about ‘a reasonable age', and they were aware of the age-related decline in the female reproductive capacity, but still the majority wanted to wait. Postponement of first birth has caused concerns, but a recent publication describing the situation in the Nordic countries indicated that fertility postponement does not always imply fewer children on an aggregate level (12). On a personal level, couples will nevertheless experience age-related infertility, if they intend to have children at an age when female fecundity is decreased.

Our impression was that men and women reflected in much the same way, with some exceptions. Few men described having children in terms of ‘A sacrifice', while this view was repeatedly voiced by the women. We believe that this finding reflects the situation for parents in many countries at present. Although more and more men share parental leave, particularly among highly educated men, it is still the norm that women in Sweden take longer breaks from their professional careers after child-birth (13). Bernhardt and Goldscheider, who analysed Swedish survey data on attitudes toward parenthood among young adults, found that both men and women generally perceived more benefits of parenthood than costs. Women with more egalitarian attitudes, however, perceived fewer benefits than did those with more traditional attitudes (14).

The experiences of social pressure to have children were more extensively described by the women. For example, some women felt they were constantly being questioned about why they do not have children, especially when living in a stable relationship. This illustrates stereotyped preconceptions about femininity and motherhood, in line with earlier research describing motherhood as the most gender-oriented female activity of all (15,16). Lampic et al. found that women were less likely than men were to accept a life without children (6).

There were some hesitations expressed when the informants discussed their longing for children in the category ‘Pros and cons of family life' and the decision to have children was seriously considered. Some of these highly educated women and men in their 30s may later realize that having children was connected with more benefits than costs, thus making it easier for them to decide on further child-bearing. Oláh and Bernharth, in an analysis of child-bearing trends in Sweden, concluded that gender equality may contribute to delayed child-bearing, on the one hand, and favour ‘recuperation' and thus lead to higher completed fertility, on the other (17). A recent report from Statistics Sweden has shown that having a third child has in fact become a new trend that is stronger in metropolitan areas. Women born in 1980 are more likely to have a third child than are women born 10 years earlier (18). The ‘child-free' informants in the present study were between 24 and 38 years old, and maybe some of the youngest of our informants will nevertheless end up with three children, if this trend continues.

Swedish official policy and ideology have actively encouraged equality between women and men in all sectors of society. The transferability of our findings should therefore be evaluated with this in mind. In order to obtain credibility in the investigation, the quality criteria for qualitative studies as outlined by Lincoln and Guba were considered (19). All authors participated in the analysis of the interviews. This approach may strengthen the conformability of the findings through the opportunity it affords researchers to supplement and challenge one another's views. The fact that all informants were highly educated, but differed in sex, age, and civil status, ensured that variations in their reflections were investigated. We believe that our findings can be transferred to similar populations and contexts. There is reason to believe that those who did not plan to have children felt less motivated to take part in the study, and therefore their perceptions are less well represented.

The interviewers (C.N. and T.T.) are midwives experienced in qualitative interviewing and family planning counselling. Being a midwife with expertise in sexual and reproductive health could be considered a strength but also a limitation, since midwives may have a certain kind of pre-understanding. Our overall impression was that both women and men liked talking about this topic; they felt relaxed and were very open-minded in the interview situation. After the interview, when we had turned off the microphone, more than half of the informants took the opportunity to ask questions concerning fertility issues. This shows that there is a need for information and counselling prior to pregnancy planning. We would like to conclude that gynaecologists and nurse midwives should help their patients to verbalize their concerns and questions related to reproduction.
